# Inhibition of Calcium‐Sensing Receptor Suppresses Malignant Behaviours of Human Hepatocellular Carcinoma

**DOI:** 10.1111/jcmm.71389

**Published:** 2026-09-29

**Authors:** Tingting Liu, Wei Xu, Qianqian Gao, N. Ngwa Adeline, Qi Yang, Dawei He, Yukai Tao, Jun Sun, Jie Gu, Haifeng Shi, Michael Aschner, Yang Ye, Jian Chen, Rongzhu Lu

**Affiliations:** ^1^ Department of Science and Technology Talents Affiliated Kunshan Hospital of Jiangsu University Kunshan Jiangsu China; ^2^ Department of Preventive Medicine and Public Health Laboratory Sciences, School of Basic Medicine and Public Health Jiangsu University Zhenjiang Jiangsu China; ^3^ Department of Pathology Yancheng Hospital of Traditional Chinese Medicine Yancheng Jiangsu China; ^4^ Department of Laboratory Medicine Affiliated Kunshan Hospital of Jiangsu University Kunshan Jiangsu China; ^5^ Department of Pathology Zhenjiang Hospital of Chinese Traditional and Western Medicine Zhenjiang Jiangsu China; ^6^ Center for Experimental Research Affiliated Kunshan Hospital of Jiangsu University Kunshan Jiangsu China; ^7^ Department of General Surgery Affiliated Kunshan Hospital of Jiangsu University Kunshan Jiangsu China; ^8^ School of Biological Science and Technology Jiangsu University Zhenjiang Jiangsu China; ^9^ Department of Molecular Pharmacology Albert Einstein College of Medicine Bronx New York USA

**Keywords:** calcium‐sensing receptor, hepatocellular carcinoma, MAPK signalling, migration, proliferation

## Abstract

Hepatocellular carcinoma (HCC) continues to pose significant worldwide health burdens due to restricted treatment alternatives and unfavourable prognosis. This investigation reveals the pivotal involvement of calcium‐sensing receptor (CaSR) in HCC advancement and its potential as a promising therapeutic target. Through pharmacological blockade with NPS‐2143 and genetic knockdown using siRNA, we found that CaSR inhibition substantially reduced HCC cell proliferation, migration, and invasion, while promoting apoptotic cell death in HepG2 and SMMC‐7721 cells. At the molecular level, CaSR interference triggered p38 MAPK activation, while inhibiting the ERK1/2 cascade, resulting in diminished expression of proliferation indicators (PCNA), metastatic markers (MMP‐2/9), and cell survival proteins (Bcl‐2), concurrent with elevated levels of apoptosis mediators (Bax, cleaved caspase‐3). In vivo, NPS‐2143 significantly suppressed xenograft tumour growth. Western blotting of xenograft tumour tissues further showed increased p‐p38/p38 and Bax levels and decreased p‐ERK/ERK, MMP‐9, and Bcl‐2 levels, consistent with the in vitro findings. Additional validation in a male nude mouse xenograft model using Huh7 cells further supported the anti‐tumour effect of NPS‐2143 in another independent HCC model. These findings suggest that CaSR facilitates malignant HCC cebehaviours through MAPK signalling and may represent a potential therapeutic target for HCC treatment.

## 1 Introduction

Calcium‐sensing receptor (CaSR) was first cloned from bovine parathyroid glands [1] and plays a vital role in maintaining calcium (Ca^2+^) homeostasis [2]. In recent years, the structure of CaSR has been gradually revealed [3], and its function is worthy of further study. Although the liver is not a calciotropic organ, Ca^2+^ is an important regulator of glucose and lipid metabolism [4], bile secretion [5], and mitochondrial activity [4], as well as cell movement, growth, differentiation, and apoptosis in liver [6]. It has been shown that alterations in the intracellular levels of Ca^2+^ in hepatocytes aggravate the accumulation of lipids that can lead to the transition of non‐alcoholic fatty liver disease to liver cancer [7]. CaSR is also involved in cholestasis [8], ischemia/reperfusion [9] and insulin resistance [10] in hepatic biology and the association of CaSR genetic polymorphisms with human hepatocellular carcinoma (HCC) [11].

CaSR exhibits dual functionality in oncological contexts. In various tumours such as parathyroid tumours [12] and gastrointestinal tumours [13, 14], CaSR can inhibit tumour growth and play an anti‐cancer role. In contrast, CaSR can exert tumour‐promoting effects and stimulate cell proliferation in intrahepatic cholangiocarcinoma [15], breast cancer [16], osteosarcoma [17], and Leydig cell tumours [18]. It has been evident that the genetic polymorphism of the *CaSR*, particularly at the rs17251221 locus, affects susceptibility to HCC and treatment outcomes [11]. However, the precise mechanisms of CaSR in HCC remain incompletely characterized.

HCC is the predominant form of primary liver cancer [19], accounting for approximately 90% of cases. Liver cancer is the sixth most commonly diagnosed cancer and the third leading cause of cancer‐related death worldwide [20]. For individuals diagnosed with early‐stage liver cancer, surgical intervention remains the primary therapeutic approach [21]. Patients presenting with intermediate or advanced disease stages typically receive chemotherapy or immunotherapy regimens [22], though the clinical benefits of these interventions remain constrained [22]. Despite advances in multimodal treatment, long‐term survival remains limited in a substantial proportion of patients with HCC [23]. Given the restricted array of available therapeutic targets for HCC, identifying novel treatment strategies has become an urgent medical priority.

Mitogen‐activated protein kinases (MAPKs) play pivotal roles in cellular signalling cascades and regulate essential cancer‐related processes, including proliferation, programmed cell death, migration, invasion, and survival [24]. Studies have shown that CaSR activation promotes metastatic behaviour in renal cell carcinoma [25] and stimulates breast cancer cell proliferation through ERK1/2‐associated signalling [26], whereas it exerts antiproliferative effects and regulates differentiation in colorectal cancer [27]. Nevertheless, the precise involvement of MAPK pathways in mediating CaSR's influence on HCC proliferation and metastatic behaviour remains unclear.

Given CaSR's established pathophysiological significance in the liver, our investigation utilizes complementary experimental approaches involving cell culture systems and animal models to elucidate CaSR's mechanistic contributions to HCC progression. This dual‐methodology strategy seeks to uncover novel molecular targets for developing improved liver cancer therapies.

## 2 Materials and Methods

### 2.1 Chemicals and Reagents

High‐glucose Dulbecco's modified Eagle's medium (DMEM) was purchased from HyClone (Logan, UT, USA). Fetal bovine serum originated from Gibco Thermo Fisher Scientific (Waltham, MA, USA). Penicillin–streptomycin antibiotic solutions were offered by Gibco (Carlsbad, CA, USA). Beyotime Biotechnology (Shanghai, China) provided 4% paraformaldehyde (PFA) and DAPI nuclear staining reagent. Sigma‐Aldrich (St. Louis, MO, USA) served as the supplier for dimethyl sulfoxide (DMSO) and the cell viability indicator MTT (3‐(4,5‐dimethylthiazol‐2‐yl)‐2,5‐diphenyltetrazolium bromide), whereas NPS‐2143 was bought from Target Molecule (Boston, MA, USA). For experimental use, NPS‐2143 was initially dissolved in DMSO, with careful attention to maintaining the final DMSO concentration in cell culture media under 0.01%.

### 2.2 Antibodies

Primary antibodies against p38 MAPK, phospho‐p38 MAPK, MMP‐9, MMP‐2, cleaved caspase‐3, ERK1/2, phospho‐ERK1/2, and PCNA were purchased from Zen BioScience (Chengdu, China). Primary antibodies against CaSR, Bax, Bcl‐2, and β‐actin were purchased from Proteintech Group (Wuhan, China). The primary antibody against Ki67 was purchased from Immunoway (Suzhou, China). HRP‐conjugated goat anti‐rabbit IgG, HRP‐conjugated goat anti‐mouse IgG, and CoraLite488‐conjugated goat anti‐rabbit IgG secondary antibodies were obtained from Proteintech Group (Wuhan, China). Detailed antibody information, including target protein, supplier, catalogue number, antibody name, host species/clonality and dilution, are provided in Table S1.

### 2.3 Protocols for Cellular Cultivation

The HepG2, SMMC‐7721 and Huh7 human liver cancer cell lines were obtained from the Shanghai Cell Bank under the Chinese Academy of Sciences. These cellular cultures were grown in DMEM supplemented with 10% FBS, penicillin (100 U/mL), and streptomycin (100 μg/mL), using high‐glucose formulation. Cell incubation was performed at 37°C in a 5% CO_2_ environment with controlled 100% humidity. Experiments were initiated when cell density reached 70%–80% confluence of culture vessels.

### 2.4 Immunofluorescence Analysis

Circular coverslip‐adhered cell cultures were chemically stabilized with 4% paraformaldehyde (PFA) for a 20‐min period at 4°C, then subjected to blocking solution containing 5% bovine serum albumin (BSA) for 60 min at room temperature. After triple washing with phosphate‐buffered saline (PBS), samples were incubated with CaSR primary antibody (1:100 concentration in 5% BSA solution) for 16–18 h at refrigerated conditions. Following further PBS rinses, specimens were treated with fluorescein isothiocyanate‐conjugated goat anti‐rabbit IgG (1:100 in PBS) for 1 h at physiological temperature under a humidity‐controlled, light‐shielded environment. Post‐washing procedures included nuclear staining with 4′,6‐diamidino‐2‐phenylindole (DAPI) during a 20‐min dark incubation. Fluorescence imaging was conducted using a Zeiss Axio Observer inverted microscope (Oberkochen, Germany).

### 2.5 Quantitative Real‐Time PCR

Total RNA was isolated from HCC cells or excised xenograft tumour tissues using TRIzol reagent (Takara, Japan). Initial cDNA synthesis was carried out employing a reverse transcription kit (Vazyme, Nanjing) according to the manufacturer's protocols. Subsequent amplification utilized SYBR Green master mix (Vazyme, Nanjing) on a CFX96 thermal cycler (Bio‐Rad, USA).

Gene‐specific primers targeting *CaSR* and the *β‐actin* reference gene were custom‐designed and manufactured by Sangon Biotech (Shanghai). The oligonucleotide sequences were as follows:

*CaSR*:

Forward primer: 5′‐GCTCTTCACCAATGGCTCCT‐3′.

Reverse primer: 5′‐AGCAGGGAGAAGAGGAGGAG‐3′.

*β‐actin*:

Forward primer: 5′‐CCTAGACTTCGAGCAAGAGA‐3′.

Reverse primer: 5′‐GGAAGGAAGGCTGGAAGA‐3′.

### 2.6 Cell Proliferation Assay

The assessment of cell proliferation was conducted using the MTT colorimetric method. HCC cell lines were plated in 96‐well microplates at a density of 6 × 10^3^ cells per well and allowed to adhere for 24 h. Following exposure to varying doses of NPS‐2143 for periods of 24, 48, or 72 h, each well received 10 μL of MTT solution (0.5 mg/mL concentration) and was incubated at 37°C for 4 h. Subsequently, 150 μL of dimethyl sulfoxide (DMSO) was introduced to dissolve the formazan crystals, and optical density measurements were taken at 490 nm wavelength using a microplate spectrophotometer (manufactured by Bio‐Rad Laboratories, Hercules, California).

### 2.7 Cell Migration and Invasion Assay

Cell migration and invasion were evaluated using Transwell chambers (BD‐Falcon, USA). For invasion assays, the upper membranes were coated with Matrigel (BD Biosciences, USA), whereas uncoated inserts were used for migration assays. A suspension of 3 × 10^4^ serum‐starved cells in 200 μL serum‐free medium was loaded into the upper chamber, and 600 μL complete media containing 10% FBS was added to the lower chamber. After 6 h of incubation at 37°C, NPS‐2143 was added to the upper chamber, and the cells were cultured for an additional 24 h. The cells were then fixed with 4% paraformaldehyde and stained with 0.1% crystal violet. Migrated or invaded cells were counted in six randomly selected microscopic fields per sample. All procedures were performed with three biological replicates.

### 2.8 siRNA Transfection

CaSR‐specific siRNA oligonucleotides were commercially obtained from RiboBio (Guangzhou, China), featuring the following targeting sequence: Si‐CaSR, 5′‐GGTTACAGGCTATGATATT‐3′.

HCC cells were plated in 6‐well (5 × 10^5^ cells/well), 24‐well (2 × 10^5^ cells/well), or 96‐well (7 × 10^3^ cells/well) plates and grown until reaching 30%–50% confluency. Transfection complexes were prepared by combining Lipofectamine RNAiMAX (Invitrogen) with siRNA and serum‐free Opti‐MEM I Medium (Gibco) in specific ratios: 3 μL transfection reagent, 5 μL siRNA, and 120 μL medium for 6‐well plates; 0.75 μL reagent, 1.25 μL siRNA, and 30 μL medium for 24‐well plates; 0.15 μL reagent, 0.25 μL siRNA, and 6 μL media for 96‐well plates. Following a 15‐min incubation, the complexes were diluted with 372 μL Opti‐MEM I Medium before being introduced to the cells. After transfection, the medium was removed and substituted with antibiotic‐free complete medium for an additional 48‐h incubation period.

### 2.9 Cell Apoptosis Detection

The apoptosis evaluation of HCC cells was performed using the FITC Annexin V detection kit (BD Biosciences, California, USA). Cellular specimens were maintained in 6‐well culture plates and exposed to either NPS‐2143 or si‐CaSR treatment. Following three washes with chilled phosphate‐buffered saline (PBS), the cellular suspension was prepared in 1× binding buffer at a density of 1 × 10^6^ cells/mL.

For analysis, 100 μL of cell suspension (containing approximately 1 × 10^5^ cells) was aliquoted into 5 mL test tubes. Subsequently, 5 μL of FITC‐conjugated Annexin V and 5 μL of propidium iodide (PI) staining solution were introduced to each sample. After brief vortex mixing, the reaction mixtures were kept at room temperature under light‐proofed conditions for 15 min. An additional 400 μL of 1× binding buffer was then incorporated into each test tube prior to analysis.

Flow cytometric analysis was performed within 1 h after staining using a flow cytometer. The acquired data were analysed using FlowJo software (BD Life Sciences, Ashland, OR, USA). Apoptotic cells were defined as the sum of early apoptotic cells (Annexin V‐positive/PI‐negative) and late apoptotic cells (Annexin V‐positive/PI‐positive).

### 2.10 Mouse Xenograft Experiments

All animal procedures were approved by the Institutional Animal Care and Use Committee of Jiangsu University and were performed in accordance with the relevant institutional guidelines for animal care and use.

For the HepG2 xenograft experiment, female BALB/c nude mice aged 5–6 weeks with a body weight of 18 ± 1.5 g were obtained from the Laboratory Animal Research Center of Jiangsu University. HepG2 cells were harvested during the logarithmic growth phase, resuspended in sterile PBS, and subcutaneously injected into the flank region of each mouse at a density of 1 × 10^6 cells per mouse. Seven days after tumour cell implantation, tumour‐bearing mice were randomly divided into two groups: the PBS control group and the NPS‐2143 treatment group (*n* = 5 per group). Mice in the treatment group received intraperitoneal injection of NPS‐2143 at a dose of 10 μmol/kg every 48 h for 12 consecutive days, whereas mice in the control group received an equivalent volume of PBS through the same route and schedule.

To address the sex‐related concern and to further validate the antitumor effect of NPS‐2143 in an independent HCC xenograft model, an additional xenograft experiment was performed using male BALB/c nude mice and Huh7 cells. Male nude mice were obtained from the same animal facility and maintained under identical housing conditions. Huh7 cells were prepared as described above and subcutaneously implanted into the flank region at a density of 1 × 10^^6^ cells per mouse. Seven days after implantation, mice were randomly assigned to the PBS control group or the NPS‐2143 treatment group (*n* = 5 per group). NPS‐2143 was administered intraperitoneally at 10 μmol/kg every 48 h for 12 days, and control mice received an equivalent volume of PBS.

### 2.11 Immunohistochemical Analysis

Mouse tumour specimens were preserved in paraffin, sliced into thin sections, and subjected to heat treatment. Tissue slides underwent deparaffinization through sequential immersion in xylene and graded alcohol solutions, then rinsed with phosphate‐buffered saline. Antigen retrieval was performed using citrate buffer, followed by a 30‐min incubation at ambient temperature after peroxidase inhibition. Blocking was achieved with goat serum before applying primary antibodies targeting Ki67 and CaSR proteins for 20 min. Counterstaining was performed using haematoxylin, after which slides were dehydrated, air‐dried, and mounted with neutral balsam. Microscopic examination was conducted at 400× magnification, with representative visual fields randomly selected for image capture.

For CaSR and Ki67 immunohistochemical analysis, tumour sections were prepared from xenograft tissues collected from the corresponding PBS control and NPS‐2143‐treated groups. Sections from different groups were stained in parallel under identical experimental conditions, and images were acquired using the same microscope settings. Comparable tumour regions were selected for representative imaging and quantitative analysis.

### 2.12 Western Blotting

Cells or excised xenograft tumour tissues were lysed in RIPA buffer supplemented with 1% PMSF. Tumour tissues were homogenized on ice before centrifugation at 12,000 × g for 15 min at 4°C. Protein concentrations were determined using a BCA assay kit (Beyotime Biotechnology, Shanghai, China). Electrophoretic separation was performed on 12% SDS‐polyacrylamide gels, followed by electroblotting onto PVDF membranes (Millipore, Germany). Membrane blocking was achieved with 5% skim milk powder in TBST solution (10 mM Tris–HCl, 120 mM sodium chloride, 0.1% Tween 20, pH 7.4) for 2 h at ambient temperature. Primary antibody incubation proceeded overnight at 4°C, followed by TBST washes and one‐hour room temperature exposure to secondary antibodies. Visualization was accomplished using the MiniChemi chemiluminescence system (Sage Creation, Beijing) with subsequent densitometric analysis performed via ImageJ software (NIH, USA).

### 2.13 Statistical Analysis

Data are presented as the mean ± standard deviation (SD) and were analysed using GraphPad Prism 7.0. Comparisons between two groups were performed using a two‐tailed unpaired Student's *t*‐test, whereas comparisons among multiple groups were analysed by one‐way ANOVA followed by Tukey's post hoc test. Time‐course data were analysed by two‐way ANOVA or two‐way repeated‐measures ANOVA, as appropriate. A two‐sided *P* value < 0.05 was considered statistically significant.

## 3 Results

### 3.1 CaSR Expression Patterns in HCC Cell Lines

Immunofluorescence analysis revealed detectable CaSR protein levels in both HepG2 and SMMC‐7721 HCC cell lines (Figure 1A). Quantitative reverse transcription PCR data demonstrated comparable expression patterns for *CaSR* mRNA across these cellular models (Figure 1B). Subsequent protein quantification through immunoblotting confirmed differential CaSR expression, with SMMC‐7721 exhibiting significantly elevated protein levels relative to HepG2 (*p* < 0.05, Figure 1C,D).

**FIGURE 1:**
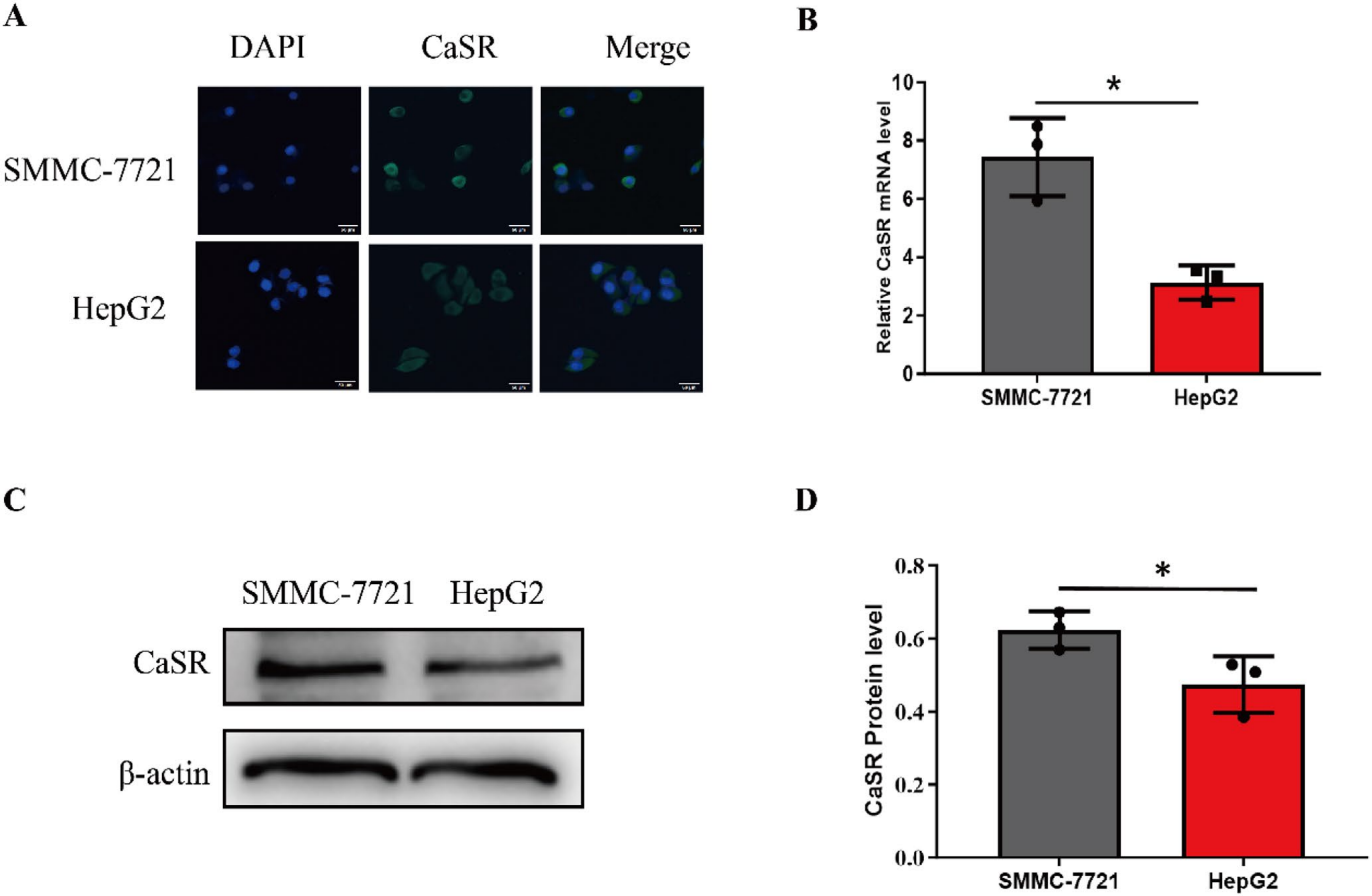
Expression of CaSR in HepG2 and SMMC‐7721 cells. (A) Representative images of immunofluorescence of CaSR protein in HepG2 and SMMC‐7721 cells. Green fluorescence indicates CaSR, and blue indicates nuclei (magnification: 400 ×; scale bars = 50 μm). (B) Levels of *CaSR* mRNA in HepG2 and SMMC‐7721 cells determined by qRT‐PCR. (C, D) The expression of CaSR protein in HepG2 and SMMC‐7721 cells (*n* = 3). *p < 0.05, using Student's *t*‐test.

### 3.2 Functional Consequences of CaSR Suppression in HCC

Pharmacological inhibition of CaSR resulted in a marked reduction of HCC cell proliferation rates. Furthermore, this intervention significantly impaired both migratory capacity and invasive potential across both tested cell lines.

To evaluate the effect of CaSR inhibition on HCC cell proliferation, HepG2 and SMMC‐7721 cells were treated with different concentrations of NPS‐2143. NPS‐2143 reduced the proliferation of both HepG2 and SMMC‐7721 cells in a concentration‐dependent manner (Figure 2A,B). Time‐course analysis further showed that NPS‐2143 markedly suppressed the proliferation of HepG2 and SMMC‐7721 cells compared with the corresponding control groups, particularly at 48 h and 72 h after treatment (Figure 2A,B). To further determine whether NPS‐2143 treatment affected CaSR expression in HCC cells, *CaSR mRNA* levels were examined by qRT‐PCR. NPS‐2143 treatment significantly reduced *CaSR* mRNA expression in both HepG2 and SMMC‐7721 cells (Figure 2C,D). Western blotting analysis further showed that NPS‐2143 decreased CaSR protein expression in both cell lines (Figure 2E,F). Consistent with the inhibition of cell proliferation, PCNA protein expression was also reduced after NPS‐2143 treatment (Figure 2E,F). These findings suggest that NPS‐2143 suppresses HCC cell proliferation and is associated with reduced CaSR expression in vitro.

**FIGURE 2:**
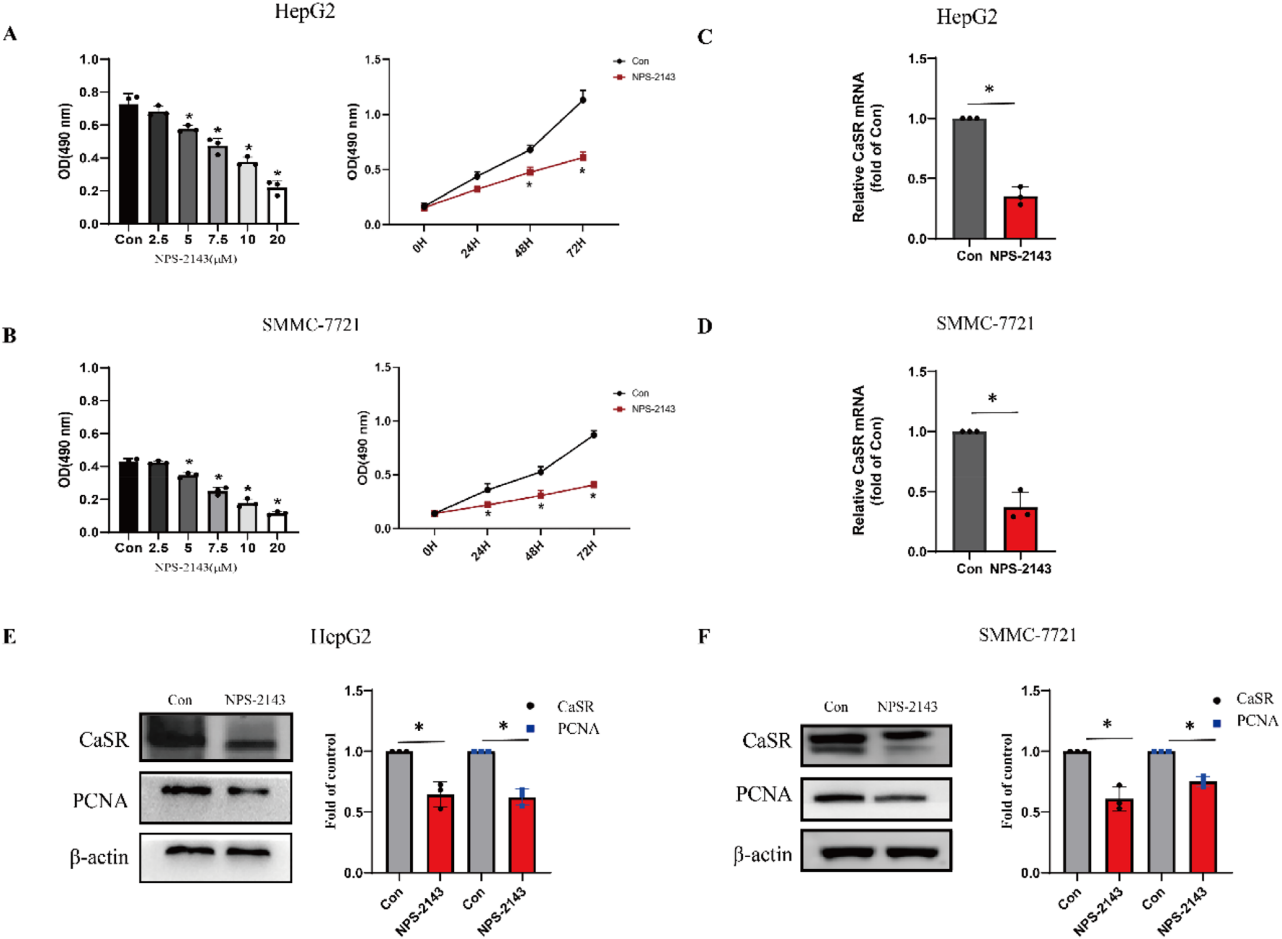
Effects of NPS‐2143 on cell proliferation and CaSR/PCNA expression in HepG2 and SMMC‐7721 cells. (A, B) HepG2 and SMMC‐7721 cells were treated with different concentrations of NPS‐2143 (2.5–20 μM) and evaluated by MTT assay (left panel). After treatment with 10 μM NPS‐2143 in HepG2 cells or 7.5 μM NPS‐2143 in SMMC‐7721 cells, cell viability was measured every 24 h (right panel). (C, D) *CaSR* mRNA expression in HepG2 and SMMC‐7721 cells after NPS‐2143 treatment was detected by qRT‐PCR. *CaSR* mRNA expression was calculated using the 2^^−ΔΔCt^ method and normalized to the Con group. (E, F) Western blotting analyses of CaSR and the proliferation‐related protein PCNA in HepG2 and SMMC‐7721 cells treated with 10 μM and 7.5 μM NPS‐2143, respectively. Proteins were normalized to β‐actin and expressed as fold of the Con group. Data are presented as mean ± SD of three independent experiments (*n* = 3). *p < 0.05 vs. Con group.

Metastasis is a major contributor to HCC recurrence and poor clinical outcomes and involves coordinated changes in cell motility, extracellular matrix remodelling, and basement membrane interactions [28, 29]. To investigate CaSR's involvement in HCC metastatic behaviour, transwell migration assays and western blot analyses were conducted. Experimental data demonstrated notable suppression of migratory and invasive properties in HepG2 and SMMC‐7721 cell lines following NPS‐2143 administration (*p* < 0.05) (Figure 3A,B). Key mediators of cellular migration, MMP‐2 and MMP‐9, exhibited decreased expression in treated HepG2 and SMMC‐7721 cells (*p* < 0.05) (Figure 3C,D). Collectively, these findings indicate that CaSR blockade attenuates HCC cell migration and invasive potential, potentially through modulation of MMP‐9 and MMP‐2 protein expression.

**FIGURE 3:**
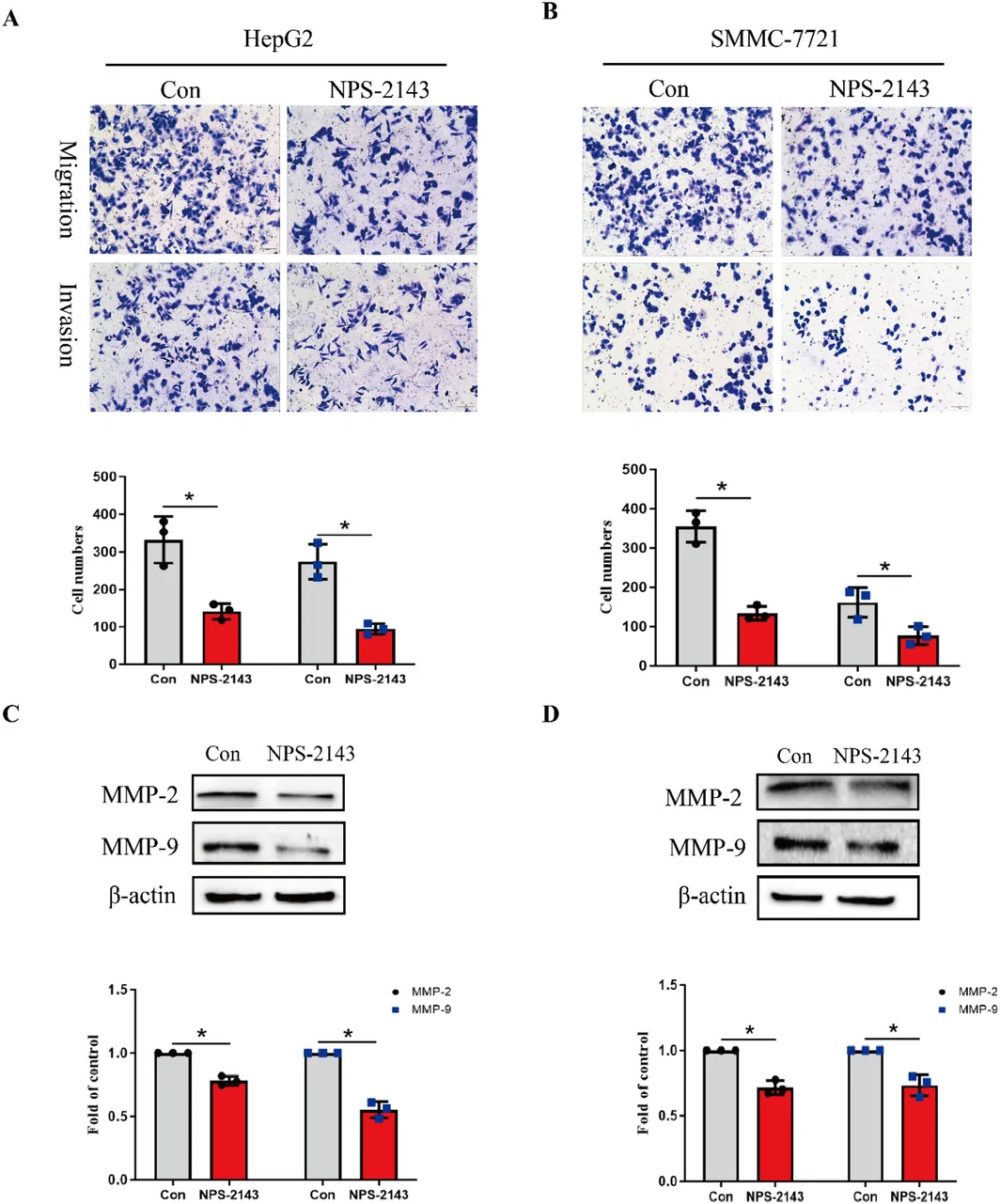
Effects of NPS‐2143 on cell migration and invasion and related protein expressions in HepG2 and SMMC‐7721 cells. Abbreviation used in the figures: “Con” denotes the control group. Unless otherwise stated, “Con” refers to untreated control cells in the in vitro experiments. (A, B) Representative images of migration and invasion assays with HepG2 and SMMC‐7721 cells treated with 10 μM and 7.5 μM NPS‐2143, respectively, for 24 h. Scale bars = 100 μm. (C, D) MMP‐2 and MMP‐9 were analysed by Western blotting in cells treated with NPS‐2143. Data are presented as mean ± SD of three independent experiments (*n* = 3). *p < 0.05 vs. Con group.

### 3.3 The Suppression of CaSR Activity Promotes Apoptotic Processes in HCC Cells

The subsequent investigation was focused on CaSR's involvement in HCC cell death pathways. Following 24‐h exposure to 10 μM NPS‐2143 in HepG2 cells and 7.5 μM in SMMC‐7721 cells, we observed a marked elevation in apoptotic cell populations (*p* < 0.05) (Figure 4A,B). To characterize the apoptotic mechanisms triggered by NPS‐2143, we performed western blot analysis of key regulatory proteins including Bax, Bcl‐2, and activated caspase 3. The immunoblot data revealed substantial downregulation of Bcl‐2 (*p* < 0.05) alongside concurrent upregulation of Bax and cleaved caspase 3 (*p* < 0.05) relative to untreated controls (Figure 4C,D). These collective findings demonstrate that pharmacological inhibition of CaSR promotes programmed cell death in HCC through coordinated regulation of apoptotic mediators.

**FIGURE 4:**
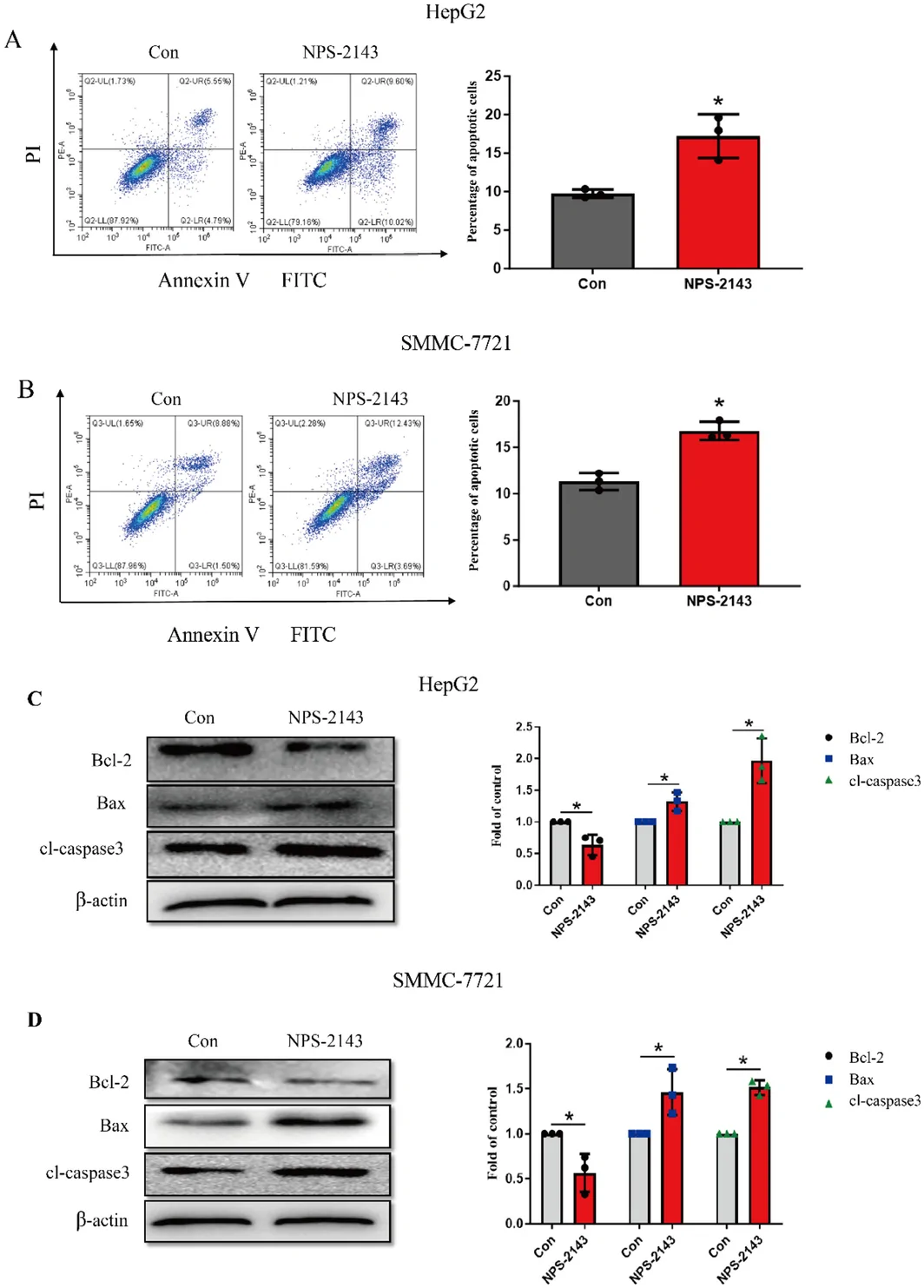
Effects of NPS‐2143 on cell apoptosis and apoptosis‐related protein expression in HepG2 and SMMC‐7721 cells. Abbreviation used in the figures: “Con” denotes the control group. Unless otherwise stated, “Con” refers to untreated control cells in the in vitro experiments. (A, B) The percentage of apoptotic cells treated with NPS‐2143 and evaluated by flow cytometry. (C, D) Bcl‐2, Bax, and cleaved caspase‐3 were analysed by Western blotting in cells after treatment with NPS‐2143. Proteins were normalized to β‐actin. Data are presented as mean ± SD of three independent experiments (*n* = 3). *p < 0.05 vs. Con group.

### 3.4 CaSR Blockade Modulates p38 and ERK Pathway Activation

Figure 5 demonstrates that NPS‐2143 administration led to a notable elevation in p38 MAPK phosphorylation (*p* < 0.05), whereas ERK MAPK phosphorylation exhibited a substantial reduction (*p* < 0.05) relative to untreated controls. Total protein levels of p38 and ERK remained unaffected following NPS‐2143 exposure. These findings imply that CaSR suppression‐mediated alterations in cellular migratory, invasive capacity, and programmed cell death likely stem from concurrent modulation of both p38 and ERK1/2 MAPK cascades.

**FIGURE 5:**
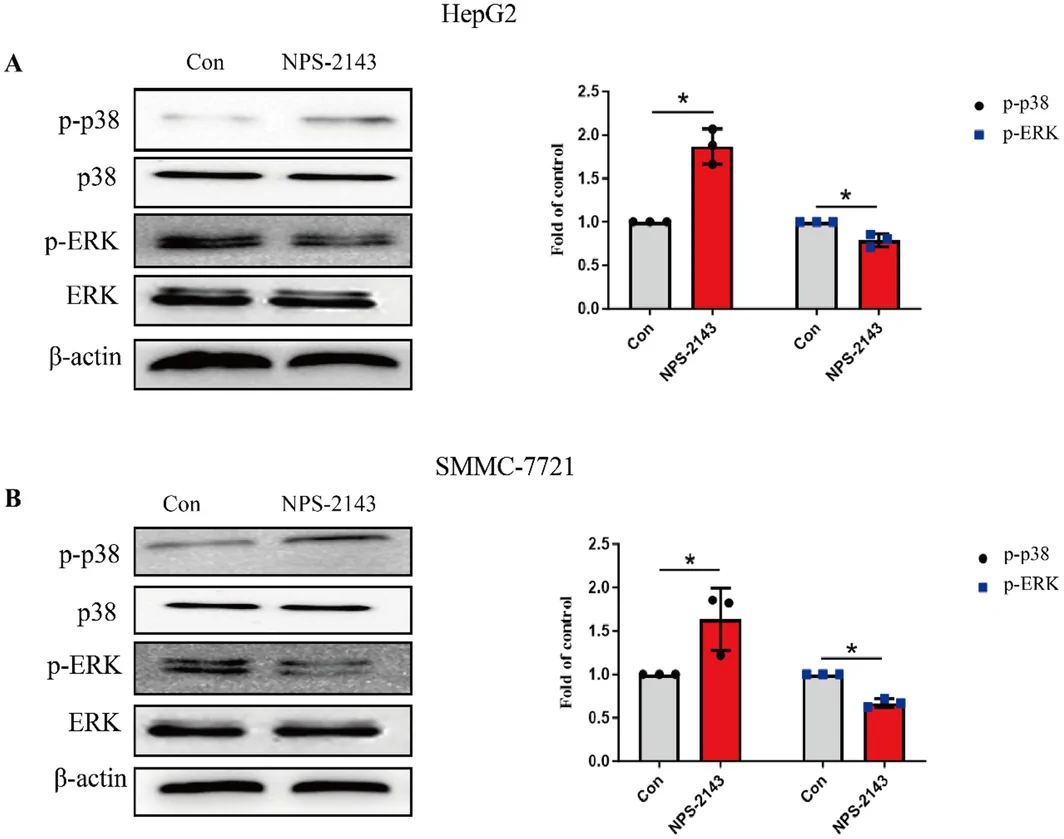
Effects of NPS‐2143 on MAPK signalling pathways in HepG2 and SMMC‐7721 cells. Abbreviation used in the figures: “Con” denotes the control group. Unless otherwise stated, “Con” refers to untreated control cells in the in vitro experiments. (A, B) Expression levels of p‐p38, p38, p‐ERK, and ERK were evaluated by Western blotting in cells treated with NPS‐2143. Phosphorylated p38 and ERK levels were normalized to their corresponding total protein levels and expressed as p‐p38/p38 and p‐ERK/ERK ratios. Total p38 and ERK proteins were normalized to β‐actin. Data are presented as mean ± SD of three independent experiments (*n* = 3). *p < 0.05 vs. Con group.

### 3.5 Genetic Silencing of CaSR Suppresses HCC Cell Growth, Migration and Invasion While Promoting Apoptosis

Experimental data reveal that CaSR depletion significantly curtails proliferative activity, migratory potential, and invasive behaviour while simultaneously increasing apoptotic rates across two distinct HCC cell models.

To investigate CaSR's involvement in HCC progression, siRNA‐mediated silencing was performed. Immunoblotting verified substantial CaSR protein reduction in HepG2 and SMMC‐7721 cells following si‐CaSR transfection (*p* < 0.05) (Figure 6). Subsequent MTT testing revealed marked growth inhibition by post‐siRNA treatment in both cellular models (*p* < 0.05) (Figure 7A,B). Migration and invasion capacities were notably impaired in CaSR‐depleted cells compared to controls, as evidenced by Transwell assay (*p* < 0.05) (Figure 7C,D). Apoptosis analysis yielded congruent findings with prior observations. Flow cytometric evaluation demonstrated elevated apoptotic cell populations in the si‐CaSR cohort (*p* < 0.05) (Figure 7E,F). Protein expression profiling revealed upregulated p‐p38 and cleaved caspase 3 alongside diminished p‐ERK in silenced cells versus negative controls (*p* < 0.05) (Figure 7G,H).

**FIGURE 6:**
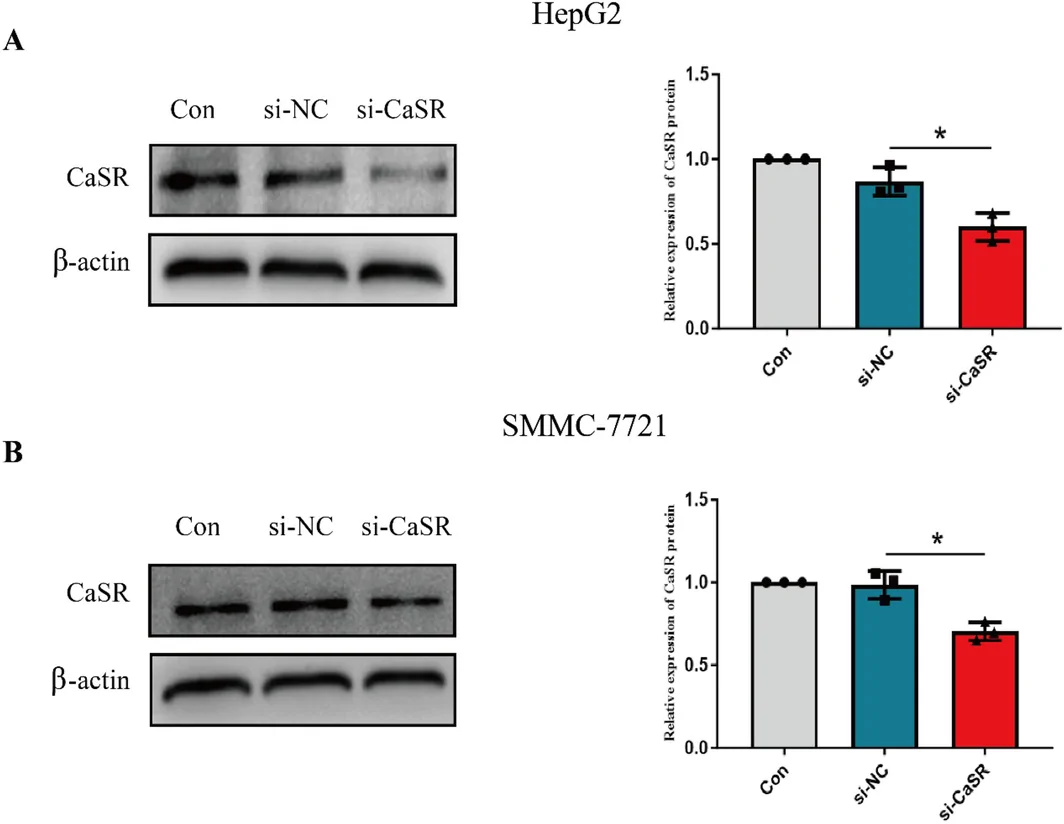
Si‐CaSR decreased CaSR protein level in HepG2 and SMMC‐7721 cells. Abbreviation used in the figures: “Con” denotes the control group. Unless otherwise stated, “Con” refers to untreated control cells in the in vitro experiments. (A, B) The protein level of CaSR in HepG2 and SMMC‐7721 cells transfected as indicated was evaluated by Western blotting. Proteins were normalized to β‐actin. Data are presented as mean ± SD of three independent experiments (*n* = 3). *p < 0.05 vs. si‐NC group.

**FIGURE 7:**
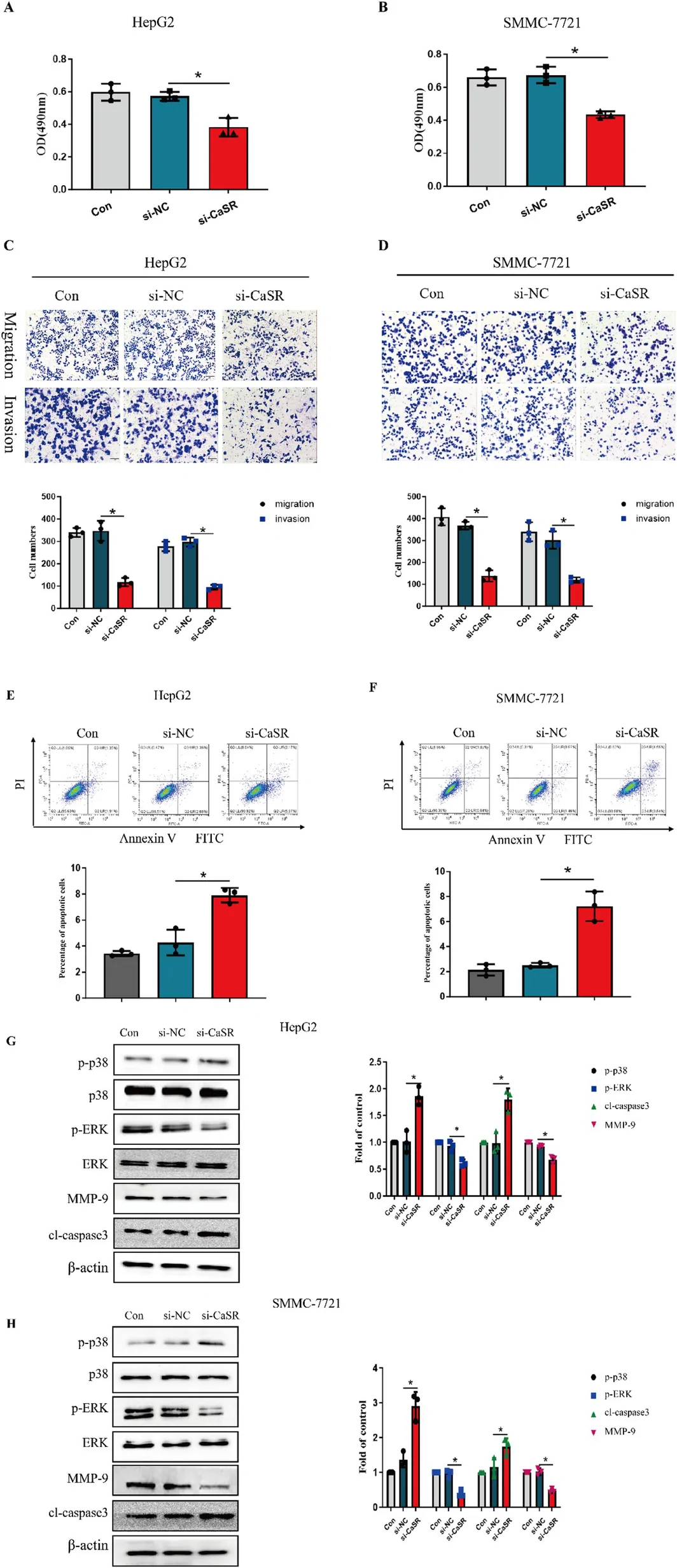
CaSR knockdown suppressed proliferation, migration, and invasion and promoted cell apoptosis in HepG2 and SMMC‐7721 cells. Abbreviation used in the figures: “Con” denotes the control group. Unless otherwise stated, “Con” refers to untreated control cells in the in vitro experiments. (A, B) CaSR knockdown inhibited cell proliferation in HepG2 and SMMC‐7721 cells. (C, D) CaSR knockdown restrained cell migration and invasion. Scale bars = 100 μm. (E, F) CaSR knockdown promoted cell apoptosis in HepG2 and SMMC‐7721 cells. (G, H) p‐p38, p‐ERK, MMP‐9 and cleaved caspase‐3 in HepG2 and SMMC‐7721 cells were evaluated by Western blotting. Proteins were normalized to β‐actin. Data are presented as mean ± SD of three independent experiments (*n* = 3). *p < 0.05 vs. si‐NC group.

### 3.6 NPS‐2143 Demonstrates Anti‐tumor Efficacy by Suppressing In Vivo Proliferation

To evaluate CaSR's involvement in tumour progression, HCC xenografts in nude mice were administered NPS‐2143 with 12‐day growth monitoring. Comparative analysis revealed diminished tumour dimensions and mass in NPS‐2143 cohorts relative to controls (Figure 8A–D). No substantial variation in body mass was observed between treatment and vehicle groups (Figure 8E). Molecular analyses showed that NPS‐2143 treatment was associated with reduced *CaSR* mRNA expression in excised xenograft tumour tissues (Figure 8F), with corresponding decreases in CaSR protein levels shown by Western blotting (Figure 8G). Because NPS‐2143 is primarily a functional antagonist of CaSR rather than a direct transcriptional inhibitor, the reduced CaSR expression observed after repeated In vivo treatment might be attributed to the treatment‐associated secondary change rather than direct transcriptional repression. Immunohistochemical evaluation further validated decreased CaSR immunoreactivity post‐NPS‐2143 administration (Figure 8H). Antiproliferative effects were quantified through Ki67 IHC, demonstrating significant reduction in tumour cell replication following NPS‐2143 exposure (Figure 8I). To further evaluate whether the in vivo anti‐tumor effect of NPS‐2143 was accompanied by molecular changes consistent with the in vitro findings, Western blotting was performed using xenograft tumour tissues. NPS‐2143 treatment increased p‐p38/p38 and Bax levels, while decreasing p‐ERK/ERK, MMP‐9, and Bcl‐2 levels (Figure 8J). These results suggest that NPS‐2143‐mediated tumour growth inhibition In vivo is associated with MAPK pathway modulation, reduced invasion‐related protein expression, and altered apoptosis‐related protein expression.

**FIGURE 8:**
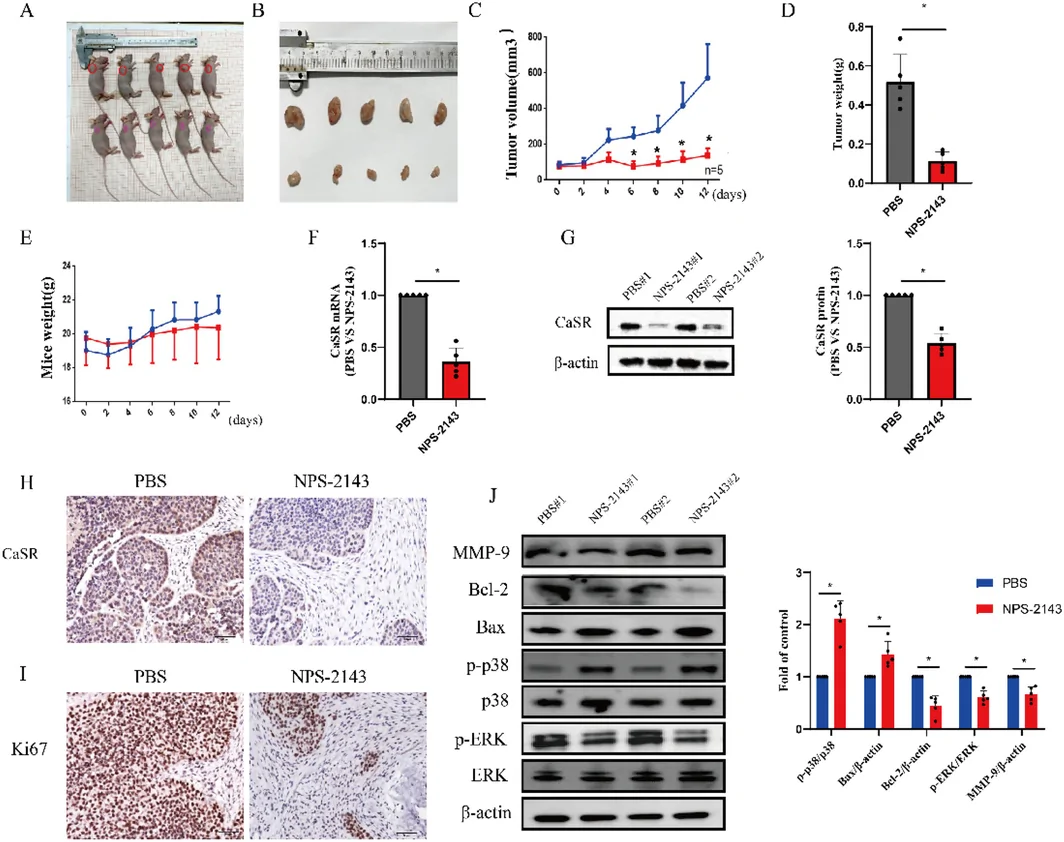
NPS‐2143 suppresses xenograft tumour growth and regulates CaSR expression, proliferation, MAPK signalling, invasion‐related protein expression, and apoptosis‐related proteins In vivo. Nude mice bearing HCC xenografts were treated with PBS or NPS‐2143. (A) Representative images of tumour‐bearing mice from the PBS control and NPS‐2143‐treated groups. (B) Representative images of excised tumours from each group. (C) Tumour volume was monitored during treatment. (D) Final tumour weight was measured after tumour collection. (E) Mouse body weight was monitored during treatment. (F) *CaSR* mRNA expression in excised xenograft tumour tissues was measured by qRT‐PCR. (G) Western blotting analysis and quantification of CaSR protein expression in xenograft tumour tissues. PBS#1 and PBS#2 represent independent tumour samples from the PBS control group, and NPS‐2143#1 and NPS‐2143#2 represent independent tumour samples from the NPS‐2143‐treated group. (H) Representative immunohistochemical staining of CaSR in tumour sections from the PBS and NPS‐2143 groups. (I) Representative immunohistochemical staining of Ki67 in tumour sections from the PBS and NPS‐2143 groups. Sections from different treatment groups were stained and imaged under identical conditions. (J) Western blotting analysis and densitometric quantification of MMP‐9, Bcl‐2, Bax, p‐p38, p38, p‐ERK, ERK, and β‐aActin in xenograft tumour tissues. MMP‐9, Bcl‐2, and Bax were normalized to β‐aActin. Phosphorylated p38 and ERK levels were normalized to their corresponding total protein levels and expressed as p‐p38/p38 and p‐ERK/ERK ratios. Bcl‐2 and Bax were used as apoptosis‐related indicators. Data are presented as mean ± SD. *p < 0.05 vs. PBS group.

### 3.7 NPS‐2143 Suppresses Huh7 Xenograft Growth in Male Nude Mice

Because HCC occurs more frequently in males and sex may influence its clinical characteristics and prognosis, we further evaluated the antitumor effect of NPS‐2143 in a male nude mouse xenograft model [30]. To provide additional validation using an independent HCC cell line, Huh7 cells were used for the male xenograft experiment. Compared with the PBS control group, NPS‐2143 treatment markedly suppressed Huh7 xenograft tumour growth, as shown by smaller tumour size, reduced final tumour weight, and slower tumour volume increase (Figure 9A–D). Representative Western blotting of xenograft tumour tissues showed lower levels of CaSR, MMP‐9, Bcl‐2, and p‐ERK, together with higher Bax levels, in the NPS‐2143‐treated group compared with the PBS control group (Figure 9E). These results suggest that NPS‐2143 suppresses HCC xenograft growth in a male mouse model derived from another HCC cell line, and this effect is accompanied by changes in CaSR‐related signalling, invasion‐related protein expression, ERK activation, and apoptosis‐related proteins.

**FIGURE 9:**
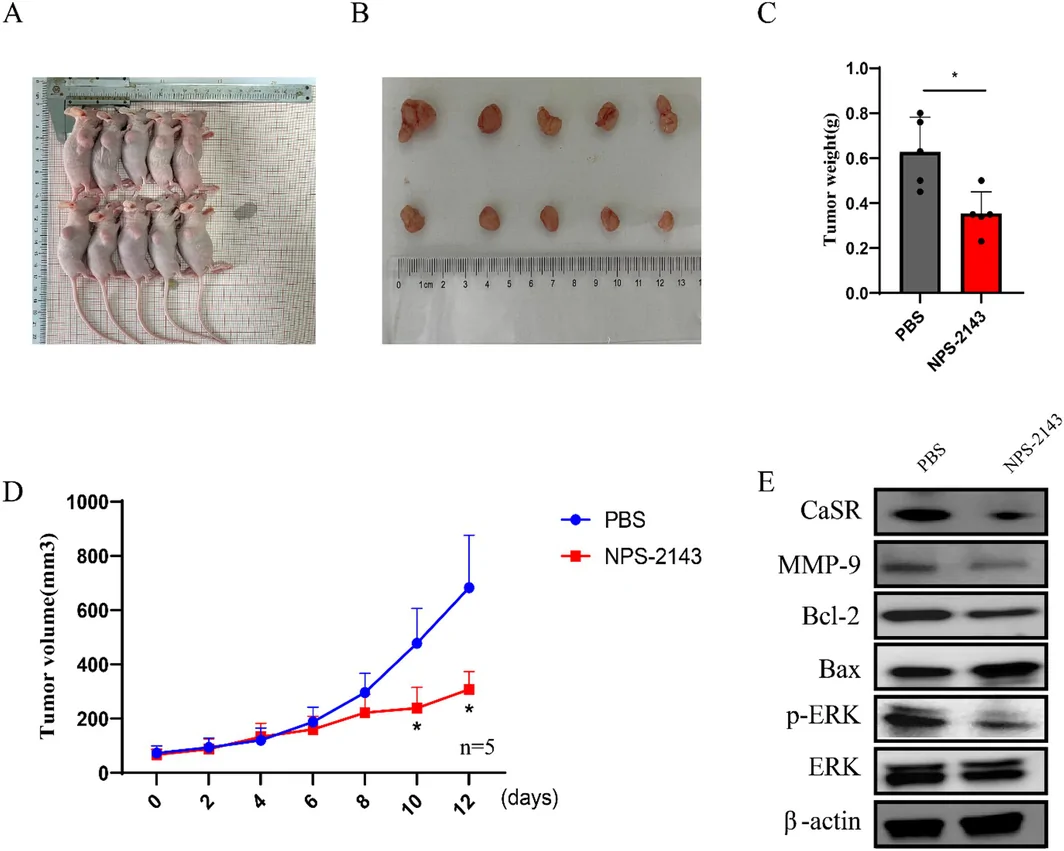
NPS‐2143 suppressed tumour growth in a male nude mouse xenograft model using Huh7 cells. Male nude mice bearing Huh7 xenografts were treated with PBS or NPS‐2143. (A) Representative images of tumour‐bearing male nude mice from the PBS control and NPS‐2143‐treated groups. (B) Representative images of excised tumours from each group. (C) Final tumour weight was measured after tumour collection. (D) Tumour volume was monitored during treatment. (E) Representative Western blotting analysis of CaSR, MMP‐9, Bcl‐2, Bax, p‐ERK, ERK, and β‐actin in xenograft tumour tissues. Bcl‐2 and Bax were used as apoptosis‐related indicators. For panels C and D, data are presented as mean ± SD. *p < 0.05 vs. PBS group.

## 4 Discussion

This study first demonstrated CaSR presence in two distinct HCC cell lines, with suppression of CaSR activity leading to reduced cellular proliferation via modulation of PCNA expression. The findings highlight CaSR's critical role in HCC advancement. Treatment with the CaSR antagonist NPS‐2143 markedly diminished aggressive HCC cell behaviours including proliferation, migration, and invasive capacity. These findings provide novel insights into CaSR's potential as a therapeutic target for HCC management.

Although SMMC‐7721 cells exhibited higher CaSR expression than HepG2 cells, SMMC‐7721 cells showed greater sensitivity to NPS‐2143, as reflected by the lower concentration required to achieve marked growth inhibition. This apparent discrepancy suggests that cellular response to CaSR antagonism is not determined solely by receptor abundance. Instead, NPS‐2143 sensitivity may also depend on receptor‐coupling efficiency, basal activity of downstream signalling pathways, and the extent to which each HCC cell line depends on CaSR‐associated survival and proliferation signalling. SMMC‐7721 cells may be more dependent on CaSR‐linked signalling networks, making them more susceptible to functional CaSR blockade despite higher basal CaSR expression. Therefore, the different responses of HepG2 and SMMC‐7721 cells likely reflect differences in signalling dependence rather than receptor expression level alone.

The introduction of two CaSR activators (CaCl_2_ and Calindol) stimulated cellular growth through modulation of the division cycle in cholangiocarcinoma cell cultures [15]. In our animal studies employing immunodeficient mouse implants, administration of NPS‐2143 demonstrated significant suppression of tumour expansion and cellular proliferation, validating its anti‐neoplastic properties. These observations align with existing research documenting NPS‐2143's growth‐inhibitory actions across various malignant cell lines and cancer subtypes such as breast cancer [26] and human retinoblastoma [31]. The experimental outcomes suggest that selective CaSR blockade could represent a new therapeutic approach for HCC management. An additional point requiring clarification is the reduced *CaSR* mRNA and protein expression observed after NPS‐2143 treatment. NPS‐2143 is primarily a CaSR antagonist/negative allosteric modulator and should not be regarded as a direct inhibitor of *CaSR* transcription [32, 33]. In the present study, *CaSR* mRNA levels were reduced after sustained NPS‐2143 exposure in HCC cells, suggesting that CaSR blockade may induce secondary feedback regulation of CaSR expression. Similar CaSR‐modulator‐associated changes in gene‐expression or signalling responses have also been reported in other tumour models. For example, pharmacological modulation of CaSR affected CaSR‐dependent gene‐expression responses in colon cancer cells [34], and NPS‐2143 treatment remodelled downstream signalling proteins, including p‐ERK1/2 and Bcl‐2, in breast cancer cells [26]. In xenograft tissues, reduced CaSR expression may additionally reflect decreased proliferation of CaSR‐positive tumour cells, altered tumour‐cell composition, or adaptive receptor regulation after repeated treatment. Therefore, we interpret the reduced CaSR expression as a treatment‐associated secondary change rather than evidence that NPS‐2143 directly represses *CaSR* transcription.

To address the potential influence of sex on the anti‐tumor effect of CaSR inhibition, we further performed an additional male nude mouse xenograft experiment using Huh7 cells. This experiment was not intended only as a gender‐control experiment, but also as an independent in vivo validation using another HCC cell line. The inhibitory effect of NPS‐2143 observed in male Huh7 xenografts supports the reproducibility of its anti‐tumor activity beyond the original female HepG2 xenograft model. Nevertheless, this additional experiment should serve as supportive validation rather than definitive evidence that the response to CaSR inhibition is independent of sex or tumour genetic background.

Apoptosis is a genetically regulated form of cellular demise governed by various protein mediators such as Bcl‐2, Bax, and Caspase 3, and plays an important role in the biology and treatment of cancer [35]. Our investigations revealed that pharmacological blockade of CaSR elevated apoptotic rates while disrupting the equilibrium of these regulatory proteins. Earlier research employing CaSR antagonists showed augmented apoptotic responses in gastric carcinoma [36] and melanoma cell lines, mediated through increased Bax, caspase‐3, and Caspase 9 expression coupled with diminished Bcl‐2 protein concentrations [37]. In intrahepatic cholangiocarcinoma cells, CaSR activation has been shown to promote cell proliferation and migration and to upregulate MMP‐2 and MMP‐9 expression, at least partly through activation of the ERK1/2 signalling pathway [15]. Nevertheless, the precise regulatory pathways through which CaSR influences these molecular targets remain incompletely characterised.

In HCC, p38 MAPK appears to exert context‐dependent effects. Activation of p38 MAPK can participate in apoptotic signalling and tumour growth suppression, whereas aberrant p38/MAPK activation may promote HCC progression in specific molecular contexts [38, 39]. Nevertheless, the exact molecular relationship between p38 and CaSR functionality in HCC pathogenesis requires further elucidation. Our experimental data revealed that pharmacological blockade of CaSR using NPS‐2143 markedly inhibited tumour cell growth while promoting programmed cell death through p38 MAPK pathway activation. Comparable biological outcomes were achieved through CaSR gene silencing in HCC cell lines. Beyond investigating p38‐mediated signalling, we additionally evaluated ERK1/2 pathway involvement. Aberrant ERK1/2 activation has been implicated in HCC proliferation, metabolic reprogramming, invasion, and therapeutic resistance [40, 41]. Our results demonstrated that CaSR suppression substantially reduced both proliferation rates and motility by downregulating phospho‐ERK1/2 expression. Earlier studies showed that CaSR stimulation promoted migration in breast cancer cells [42] and proliferation in osteosarcoma cells through ERK1/2 and PI3K–AKT signalling [17]. These observations align with existing reports showing that CaSR signalling interacts with MAPK‐related pathways to regulate cancer‐associated cellular functions, including proliferation, migration, invasion, and apoptosis [26].

Several constraints should be acknowledged in this research. Given the extensive molecular diversity of HCC, the two cellular models employed here fail to capture the full spectrum of disease variability. Expanding the experimental framework to include additional models, particularly patient‐derived xenografts, would enhance the generalizability of our conclusions. Although NPS‐2143 demonstrates specificity as a CaSR allosteric modulator, potential interactions with unintended molecular targets cannot be entirely dismissed. Complementary approaches involving CRISPR/Cas9‐mediated *CaSR* knockout could strengthen the pharmacological evidence and provide more definitive validation of CaSR's role in HCC pathogenesis.

The statistical robustness of our findings is somewhat limited by the modest animal numbers in mice experiments. More extensive trials with increased sample numbers would be necessary to verify these observations with greater confidence. Nevertheless, our preliminary data suggest therapeutic potential for CaSR modulation in two murine xenograft systems. It's important to note that the immunodeficient mouse model employed here cannot fully recapitulate the complex immunological microenvironment characteristic of human HCC. Subsequent investigations using immunocompetent animal models would be valuable for elucidating potential immune‐related mechanisms associated with CaSR blockade.

## 5 Conclusion

CaSR is expressed in HCC cell lines, and CaSR inhibition suppresses HCC cell proliferation, migration, and invasion, while promoting apoptosis‐related changes through regulation of p38 and ERK1/2 MAPK signalling pathways. In vivo, NPS‐2143 treatment reduced xenograft tumour growth, and additional male Huh7 xenograft data further supported its anti‐tumor activity in another independent HCC model. These findings suggest that CaSR may represent a potential therapeutic target for HCC. Further studies are required to clarify the detailed mechanisms and evaluate the translational potential of CaSR blockade for HCC treatment.

## Author Contributions

**Tingting Liu:** conceptualization, methodology, software, data curation, investigation, validation, funding acquisition, project administration, writing – original draft, writing – review and editing. **Wei Xu:** data curation, investigation, writing – review and editing. **Qianqian Gao:** project administration, writing – review and editing, data curation, funding acquisition. **N. Ngwa Adeline:** writing – review and editing. **Qi Yang:** writing – review and editing. **Dawei He:** writing – review and editing. **Yukai Tao:** writing – review and editing. **Jun Sun:** writing – review and editing. **Jie Gu:** writing – review and editing. **Haifeng Shi:** writing – review and editing. **Michael Aschner:** writing – review and editing. **Yang Ye:** writing – review and editing. **Jian Chen:** writing – review and editing. **Rongzhu Lu:** conceptualization, methodology, writing – review and editing, visualization, project administration, supervision.

## Funding

This study was supported in part by the Kunshan Key R&D Program (Social Development) (No. KS2423, No. KS2404), Kunshan First People's Hospital Health and Medical Technology Innovation Special Project (KETDCX202416), the Natural Science Foundation of NJUCM (NO. XZR 2023097) and by grants from the scientific research project of Jiangsu Provincial Health Commission (No. Z2022076), Science and Technology Program of Suzhou (SYWD2025227 and SYW2025074), and the Nantong University Clinical Medicine Special Support Project (No. 2025JY062).

## Ethics Statement

The experimental procedures involving animals received approval from the Animal Ethics Committee at Jiangsu University (Approval No. UJS‐IACUC‐2020052101). All investigations strictly followed applicable regulatory guidelines and institutional protocols governing animal research.

## Conflicts of Interest

The authors declare no conflicts of interest.

## Supporting information


**Table S1:** Antibodies used in this study.

## Data Availability

The data that support the findings of this study are available from the corresponding author upon reasonable request.

## References

[1] E. M. Brown , G. Gamba , D. Riccardi , et al., “Cloning and Characterization of an Extracellular Ca2+−Sensing Receptor From Bovine Parathyroid,” Nature 366, no. 6455 (1993): 575–580, 10.1038/366575a0. 8255296

[2] L. Tian , C. Andrews , Q. Yan , and J. J. Yang , “Molecular Regulation of Calcium‐Sensing Receptor (CaSR)‐Mediated Signaling,” Chronic Diseases and Translational Medicine 10, no. 3 (2024): 167–194, 10.1002/cdt3.123. PMC11252437 39027195

[3] H. Zuo , J. Park , A. Frangaj , et al., “Promiscuous G‐Protein Activation by the Calcium‐Sensing Receptor,” Nature 629, no. 8011 (2024): 481–488, 10.1038/s41586-024-07331-1. PMC11844898 38632411

[4] T. E. LaMoia , B. T. Hubbard , M. T. Guerra , et al., “Cytosolic Calcium Regulates Hepatic Mitochondrial Oxidation, Intrahepatic Lipolysis, and Gluconeogenesis via CaMKII Activation,” Cell Metabolism 36, no. 10 (2024): 2329–2340.e4, 10.1016/j.cmet.2024.07.016. PMC11446666 39153480

[5] L. Canaff , J. L. Petit , M. Kisiel , et al., “Extracellular Calcium‐Sensing Receptor Is Expressed in Rat Hepatocytes: Coupling to Intracellular Calcium Mobilization and Stimulation of Bile Flow,” Journal of Biological Chemistry 276, no. 6 (2001): 4070–4079, 10.1074/jbc.M009317200. 11071898

[6] T. Liu , W. Xu , Q. Gao , et al., “Calcium‐Sensing Receptor: A Potential Target for Liver Health and Diseases,” Journal of Translational Medicine 23, no. 1 (2025): 1101, 10.1186/s12967-025-07182-y. PMC12522512 41094551

[7] E. S. Ali , G. Y. Rychkov , and G. J. Barritt , “Deranged Hepatocyte Intracellular Ca2+ Homeostasis and the Progression of Nonalcoholic Fatty Liver Disease to Hepatocellular Carcinoma,” Cell Calcium 82 (2019): 102057, 10.1016/j.ceca.2019.102057. 31401389

[8] H. Qin , L. L. Zhang , X. L. Xiong , et al., “Li‐Dan‐He‐Ji Improves Infantile Cholestasis Hepatopathy Through Inhibiting Calcium‐Sensing Receptor‐Mediated Hepatocyte Apoptosis,” Frontiers in Pharmacology 11 (2020): 156, 10.3389/fphar.2020.00156. PMC7059769 32180721

[9] F. Paquot , J. Huart , J. O. Defraigne , J. M. Krzesinski , and F. Jouret , “Implications of the Calcium‐Sensing Receptor in Ischemia/Reperfusion,” Acta Cardiologica 72, no. 2 (2017): 125–131, 10.1080/00015385.2017.1291136. 28597792

[10] S. Guo , T. Yan , L. Shi , et al., “Matrine, as a CaSR Agonist Promotes Intestinal GLP‐1 Secretion and Improves Insulin Resistance in Diabetes Mellitus,” Phytomedicine 84 (2021): 153507, 10.1016/j.phymed.2021.153507. 33636577

[11] Q. Tang , Y. Zhao , Y. Wang , et al., “A Genetic Variant (rs17251221) in the Calcium‐Sensing Receptor Relates to Hepatocellular Carcinoma Susceptibility and Clinical Outcome Treated by Transcatheter Hepatic Arterial Chemoembolization (TACE) Therapy,” Medical Oncology 31, no. 11 (2014): 267, 10.1007/s12032-014-0267-2. 25270285

[12] P. Singh , S. K. Bhadada , D. Dahiya , et al., “Reduced Calcium‐Sensing Receptor (CaSR) Expression Is Epigenetically Deregulated in Parathyroid Adenomas,” Journal of Clinical Endocrinology and Metabolism 105, no. 9 (2020): 3015–3024, 10.1210/clinem/dgaa419. PMC7500582 32609827

[13] S. Khan , S. N. Mosvi , S. Vohra , and N. K. Poddar , “Implication of Calcium Supplementations in Health and Diseases With Special Focus on Colorectal Cancer,” Critical Reviews in Clinical Laboratory Sciences 61, no. 6 (2024): 496–509, 10.1080/10408363.2024.2322565. 38456354

[14] A. Aggarwal , M. Prinz‐Wohlgenannt , C. Gröschel , et al., “The Calcium‐Sensing Receptor Suppresses Epithelial‐To‐Mesenchymal Transition and Stem Cell‐Like Phenotype in the Colon,” Molecular Cancer 14 (2015): 61, 10.1186/s12943-015-0330-4. PMC4405849 25879211

[15] Y. Fang , L. Liu , S. Liu , et al., “Calcium‐Sensing Receptor Promotes Tumor Proliferation and Migration in Human Intrahepatic Cholangiocarcinoma by Targeting ERK Signaling Pathway,” European Journal of Pharmacology 872 (2020): 172915, 10.1016/j.ejphar.2020.172915. 31926993

[16] Y. El Hiani , A. Ahidouch , V. Lehen'kyi , et al., “Extracellular Signal‐Regulated Kinases 1 and 2 and TRPC1 Channels Are Required for Calcium‐Sensing Receptor‐Stimulated MCF‐7 Breast Cancer Cell Proliferation,” Cellular Physiology and Biochemistry 23, no. 4–6 (2009): 335–346, 10.1159/000218179. 19471101

[17] W. Zhao , Z. Zhang , L. Zheng , et al., “Calcium‐Sensing Receptor Activating ERK1/2 and PI3K‐Akt Pathways to Induce the Proliferation of Osteosarcoma Cells,” Clinical and Experimental Pharmacology & Physiology 47, no. 3 (2020): 517–519, 10.1111/1440-1681.13209. 31705676

[18] J. Tfelt‐Hansen , N. Chattopadhyay , S. Yano , et al., “Calcium‐Sensing Receptor Induces Proliferation Through p38 Mitogen‐Activated Protein Kinase and Phosphatidylinositol 3‐Kinase but Not Extracellularly Regulated Kinase in a Model of Humoral Hypercalcemia of Malignancy,” Endocrinology 145, no. 3 (2004): 1211–1217, 10.1210/en.2003-0749. 14645111

[19] European Association for the Study of the Liver , “EASL Clinical Practice Guidelines on the Management of Hepatocellular Carcinoma,” Journal of Hepatology 82, no. 2 (2025): 315–374, 10.1016/j.jhep.2024.08.028. 39690085

[20] F. Bray , M. Laversanne , H. Sung , et al., “Global Cancer Statistics 2022: GLOBOCAN Estimates of Incidence and Mortality Worldwide for 36 Cancers in 185 Countries,” CA: A Cancer Journal for Clinicians 74, no. 3 (2024): 229–263, 10.3322/caac.21834. 38572751

[21] Y. T. Chan , C. Zhang , J. Wu , et al., “Biomarkers for Diagnosis and Therapeutic Options in Hepatocellular Carcinoma,” Molecular Cancer 23, no. 1 (2024): 189, 10.1186/s12943-024-02101-z. PMC11378508 39242496

[22] J. Bruix , S. L. Chan , P. R. Galle , L. Rimassa , and B. Sangro , “Systemic Treatment of Hepatocellular Carcinoma: An EASL Position Paper,” Journal of Hepatology 75, no. 4 (2021): 960–974, 10.1016/j.jhep.2021.07.004. 34256065

[23] S. S. Kim , J. Lee , S. B. Ahn , et al., “Clinical Course and Prognosis of Long‐Term Survivors of Hepatocellular Carcinoma,” Alimentary Pharmacology & Therapeutics 61, no. 8 (2025): 1333–1342, 10.1111/apt.70004. 39955712

[24] M. E. Bahar , H. J. Kim , and D. R. Kim , “Targeting the RAS/RAF/MAPK Pathway for Cancer Therapy: From Mechanism to Clinical Studies,” Signal Transduction and Targeted Therapy 8, no. 1 (2023): 455, 10.1038/s41392-023-01705-z. PMC10725898 38105263

[25] S. Frees , I. Breuksch , T. Haber , et al., “Calcium‐Sensing Receptor (CaSR) Promotes Development of Bone Metastasis in Renal Cell Carcinoma,” Oncotarget 9, no. 21 (2018): 15766–15779, 10.18632/oncotarget.24607. PMC5884663 29644008

[26] M. A. Y. Alqudah , M. Azaizeh , A. Zayed , and L. Asaad , “Calcium‐Sensing Receptor Antagonist NPS‐2143 Inhibits Breast Cancer Cell Proliferation, Migration and Invasion via Downregulation of p‐ERK1/2, Bcl‐2 and Integrin β1 and Induces Caspase‐3/7 Activation,” Advanced Pharmaceutical Bulletin 12, no. 2 (2022): 383–388, 10.34172/apb.2022.037. PMC9106957 35620345

[27] A. Aggarwal , M. Prinz‐Wohlgenannt , S. Tennakoon , et al., “The Calcium‐Sensing Receptor: A Promising Target for Prevention of Colorectal Cancer,” Biochimica et Biophysica Acta‐Molecular Cell Research 1853, no. 9 (2015): 2158–2167, 10.1016/j.bbamcr.2015.02.011. PMC4549785 25701758

[28] K. Wei , C. Peng , Y. Ou , et al., “Decoding Hepatocellular Carcinoma Metastasis: Molecular Mechanisms, Targeted Therapies, and Potential Biomarkers,” Current Issues in Molecular Biology 47, no. 4 (2025): 263, 10.3390/cimb47040263. PMC12025613 40699663

[29] S. Wei , J. Tan , X. Huang , et al., “Metastasis and Basement Membrane‐Related Signature Enhances Hepatocellular Carcinoma Prognosis and Diagnosis by Integrating Single‐Cell RNA Sequencing Analysis and Immune Microenvironment Assessment,” Journal of Translational Medicine 22, no. 1 (2024): 711, 10.1186/s12967-024-05493-0. PMC11293133 39085893

[30] J. Chen , X. Wang , and W. Ye , “Prognostic Analysis of Sex and Age in Hepatocellular Carcinoma: A SEER Study,” European Journal of Gastroenterology & Hepatology 36, no. 5 (2024): 646–651, 10.1097/meg.0000000000002745. PMC10965129 38555602

[31] C. Zhang , T. Yu , G. Sun , and M. Li , “NPS‐2143 Suppresses Malignant Phenotypes of Retinoblastoma Cells Involved in Regulating NF‐κB Pathway,” Scientific Reports 15, no. 1 (2025): 16209, 10.1038/s41598-025-00783-z. PMC12064661 40346093

[32] F. M. Hannan , G. V. Walls , V. N. Babinsky , et al., “The Calcilytic Agent NPS 2143 Rectifies Hypocalcemia in a Mouse Model With an Activating Calcium‐Sensing Receptor (CaSR) Mutation: Relevance to Autosomal Dominant Hypocalcemia Type 1 (ADH1),” Endocrinology 156, no. 9 (2015): 3114–3121, 10.1210/en.2015-1269. PMC4541614 26052899

[33] T. Wen , Z. Wang , X. Chen , et al., “Structural Basis for Activation and Allosteric Modulation of Full‐Length Calcium‐Sensing Receptor,” Science Advances 7, no. 23 (2021): eabg1483, 10.1126/sciadv.abg1483. PMC8177707 34088669

[34] M. Schepelmann , N. Kupper , M. Sladczyk , et al., “Stereo‐Specific Modulation of the Extracellular Calcium‐Sensing Receptor in Colon Cancer Cells,” International Journal of Molecular Sciences 22, no. 18 (2021): 10124, 10.3390/ijms221810124. PMC8464956 34576291

[35] A. Moyer , K. Tanaka , and E. H. Cheng , “Apoptosis in Cancer Biology and Therapy,” Annual Review of Pathology 20, no. 1 (2025): 303–328, 10.1146/annurev-pathmechdis-051222-115023. 39854189

[36] Z. L. Zhang , Z. R. Li , J. S. Li , and S. R. Wang , “Calcium‐Sensing Receptor Antagonist NPS‐2143 Suppresses Proliferation and Invasion of Gastric Cancer Cells,” Cancer Gene Therapy 27, no. 7–8 (2020): 548–557, 10.1038/s41417-019-0128-4. 31391530

[37] S. Wang , L. Qiu , H. Song , and N. Dang , “NPS‐2143 (Hydrochloride) Inhibits Melanoma Cancer Cell Proliferation and Induces Autophagy and Apoptosis,” Medecine Sciences 34, no. Focus issue F1 (2018): 87–93, 10.1051/medsci/201834f115. 30403181

[38] W. J. Lu , M. S. Chua , and S. K. So , “Suppression of ATAD2 Inhibits Hepatocellular Carcinoma Progression Through Activation of p53‐ and p38‐Mediated Apoptotic Signaling,” Oncotarget 6, no. 39 (2015): 41722–41735, 10.18632/oncotarget.6152. PMC4747184 26497681

[39] X. Zhang , K. Dong , J. Zhang , et al., “GNB1 Promotes Hepatocellular Carcinoma Progression by Targeting BAG2 to Activate P38/MAPK Signaling,” Cancer Science 114, no. 5 (2023): 2001–2013, 10.1111/cas.15741. PMC10154882 36718954

[40] T. Huang , S. Ge , W. Huang , et al., “AIBP Promotes Cell Proliferation and Migration Through the ERK1/2‐MAPK Signaling Pathway in Hepatocellular Carcinoma,” Translational Cancer Research 13, no. 8 (2024): 4028–4041, 10.21037/tcr-23-2101. PMC11384315 39262469

[41] K. He , M. Xie , W. Hong , et al., “CENPL Accelerates Cell Proliferation, Cell Cycle, Apoptosis, and Glycolysis via the MEK1/2‐ERK1/2 Pathway in Hepatocellular Carcinoma,” International Journal of Biochemistry & Cell Biology 166 (2024): 106481, 10.1016/j.biocel.2023.106481. 37914022

[42] Z. Saidak , C. Boudot , R. Abdoune , et al., “Extracellular Calcium Promotes the Migration of Breast Cancer Cells Through the Activation of the Calcium‐Sensing Receptor,” Experimental Cell Research 315, no. 12 (2009): 2072–2080, 10.1016/j.yexcr.2009.03.003. 19285978

