# Supplementary material for: Inhibition of Calcium‐Sensing Receptor Suppresses Malignant Behaviours of Human Hepatocellular Carcinoma
Source: J Cell Mol Med. 2026 Sep 29;30(19):e71389. doi: 10.1111/jcmm.71389 (PMC13624293; doi:10.1111/jcmm.71389)
Supplement: Supplementary file 1 [file JCMM-30-e71389-s001.docx]

Supplementary Table S1. Antibodies used in this study.

| **Target** | **Supplier** | **Catalog No.** | **Antibody name** | **Host / Type** | **Dilution** |
| --- | --- | --- | --- | --- | --- |
| p38 MAPK | Zen BioScience | R25239 | p38 MAPK Rabbit mAb | Rabbit monoclonal | 1:1000 |
| p-p38 MAPK | Zen BioScience | 310091 | Phospho-p38 MAPK (Thr180/Tyr182) Rabbit pAb | Rabbit polyclonal | 1:1000 |
| MMP-9 | Zen BioScience | R380831 | MMP9 Rabbit mAb | Rabbit monoclonal | 1:1000 |
| MMP-2 | Zen BioScience | R380817 | MMP2 Rabbit mAb | Rabbit monoclonal | 1:1000 |
| cleaved caspase-3 | Zen BioScience | 341034 | Cleaved-Caspase 3 p17 Rabbit pAb | Rabbit polyclonal | 1:1000 |
| ERK1/2 | Zen BioScience | 343830 | ERK1/2 Rabbit pAb | Rabbit polyclonal | 1:1000 |
| PCNA | Zen BioScience | R25294 | PCNA Rabbit mAb | Rabbit monoclonal | 1:1000 |
| p-ERK1/2 | Zen BioScience | 310065 | Phospho-ERK1/2 (Tyr204/Tyr187) Rabbit pAb | Rabbit polyclonal | 1:1000 |
| β-actin | Proteintech | 66009-1-Ig | Beta Actin Monoclonal antibody | Mouse monoclonal / IgG2b | 1:10000 |
| CaSR | Proteintech | 86319-1-RR | CASR Recombinant monoclonal antibody | Rabbit recombinant monoclonal | WB: 1:1000; IF/IHC: 1:100 |
| Bax | Proteintech | 50599-2-Ig | Bax Polyclonal antibody | Rabbit polyclonal | 1:1000 |
| Bcl-2 | Proteintech | 12789-1-AP | Bcl-2 Polyclonal antibody | Rabbit polyclonal | 1:1000 |
| HRP-conjugated secondary antibody | Proteintech | SA00001-2 | HRP-conjugated Goat Anti-Rabbit IgG(H+L) | Goat anti-rabbit | 1:5000 |
| HRP-conjugated secondary antibody | Proteintech | SA00001-1 | HRP-conjugated Goat Anti-Mouse IgG(H+L) | Goat anti-mouse | 1:5000 |
| Fluorescent secondary antibody | Proteintech | SA00013-2 | CoraLite488-conjugated Goat Anti-Rabbit IgG(H+L) | Goat anti-rabbit | 1:100 |
| Ki-67 | immunoway | YM8189 | Ki-67 (PT0321R) PT™ Rabbit mAb | Rabbit polyclonal | IHC 1:200 |
